# Repurposing mebendazole against triple-negative breast cancer CNS metastasis

**DOI:** 10.1007/s11060-024-04654-x

**Published:** 2024-04-02

**Authors:** Adrian J. Rodrigues, Sophia B. Chernikova, Yuelong Wang, Thy T. H. Trinh, David E. Solow-Cordero, Ludmila Alexandrova, Kerriann M. Casey, Elizabeth Alli, Abhishek Aggarwal, Tyler Quill, Ashley K. Koegel, Brian J. Feldman, James M. Ford, Melanie Hayden-Gephart

**Affiliations:** 1 Department of Neurosurgery, Stanford School of Medicine, Stanford, CA 94305 USA; 2 Department of Neurosurgery, West China Hospital, Sichuan University, Chengdu, China; 3 High-Throughput Screening Knowledge Center, Sarafan ChEM-H, Stanford, CA 94305 USA; 4 Vincent Coates Foundation Mass Spectrometry Laboratory, Stanford University, Stanford, CA 94305 USA; 5 Department of Comparative Medicine, Stanford University School of Medicine, Stanford, CA 94305 USA; 6 Department of Cancer Biology, Wake Forest University School of Medicine, Winston-Salem, NC 27109 USA; 7 Department of Materials Science and Engineering, Stanford University, Stanford, CA 94305 USA; 8 Department of Pediatric Hematology-Oncology, University of California, San Francisco, CA 94143 USA; 9 Department of Pediatrics, University of California, San Francisco, CA 94143 USA; 10 Department of Medicine (Oncology), Stanford School of Medicine, Stanford, CA 94305 USA; 11 Present Address: Department of Neurosurgery, Massachusetts General Hospital, Boston, MA 02114 USA; 12 Department of Pediatrics, Stanford University, Stanford, CA USA

**Keywords:** Breast cancer, Leptomeningeal disease, Drug repurposing, Mebendazole

## Abstract

**Purpose:**

Triple-negative breast cancer (TNBC) often metastasizes to the central nervous system (CNS) and has the highest propensity among breast cancer subtypes to develop leptomeningeal disease (LMD). LMD is a spread of cancer into leptomeningeal space that speeds up the disease progression and severely aggravates the prognosis. LMD has limited treatment options. We sought to test whether the common anti-helminthic drug mebendazole (MBZ) may be effective against murine TNBC LMD.

**Methods:**

A small-molecule screen involving TNBC cell lines identified benzimidazoles as potential therapeutic agents for further study. *In vitro* migration assays were used to evaluate cell migration capacity and the effect of MBZ. For *in vivo* testing, CNS metastasis was introduced into BALB/c athymic nude mice through internal carotid artery injections of brain-tropic MDA-MB-231-BR or MCF7-BR cells. Tumor growth and spread was monitored by bioluminescence imaging and immunohistochemistry. MBZ was given orally at 50 and 100 mg/kg doses. MBZ bioavailability was assayed by mass spectrometry.

**Results:**

Bioinformatic analysis and migration assays revealed higher migratory capacity of TNBC compared to other breast cancer subtypes. MBZ effectively slowed down migration of TNBC cell line MDA-MB-231 and its brain tropic derivative MDA-MB-231-BR. In animal studies, MBZ reduced leptomeningeal spread, and extended survival in brain metastasis model produced by MDA-MB-231-BR cells. MBZ did not have an effect in the non-migratory MCF7-BR model.

**Conclusions:**

We demonstrated that MBZ is a safe and effective oral agent in an animal model of TNBC CNS metastasis. Our findings are concordant with previous efforts involving MBZ and CNS pathology and support the drug’s potential utility to slow down leptomeningeal spread.

**Supplementary Information:**

The online version contains supplementary material available at 10.1007/s11060-024-04654-x.

## Introduction

Triple-negative breast cancer (TNBC) is an aggressive breast cancer subtype that metastasizes to the central nervous system (CNS) in up to 50% of affected patients [1]. Once disseminated to CNS, TNBC carries poor prognosis, with limited treatment options [2], and a median survival of only 5 months [1]. Patient’s prognosis is severely aggravated when cancer spreads to leptomeninges and cerebral spinal fluid (CSF), developing leptomeningeal disease (LMD, also known as leptomeningeal carcinomatosis, neoplastic meningitis, or carcinomatous meningitis) [3]. While LMD is documented to occur in a minority of breast cancers, its incidence is increasing [4, 5]. Among breast cancer subtypes, TNBC accounts for the shortest time between primary diagnosis and CNS metastasis [6]. Rapid metastatic dissemination of TNBC is likely based on its high migratory potential [7]. Current standard-of-care for LMD involves systemic and intrathecal chemotherapy with- or without palliative whole brain radiation, with limited efficacy [8].

Given the paucity of effective treatments and challenges with identifying and approving new drugs for relatively small patient populations, recent work has begun to focus on repurposing previously approved pharmaceutical agents. This approach has two main advantages over the *de novo* development: substantially reduced costs and an accelerated time-to-patient use [9]. In the field of neuro-oncology, drug repurposing is of particular interest due to the high cost of new therapies and the limited effectiveness of available treatments [3, 4, 8].

In the study of CNS tumors, drug repurposing efforts have highlighted the benzimidazole anti-helminthic class, including mebendazole (MBZ), albendazole (ABZ), and fenbendazole (FBZ) [10, 11]. This drug class is widely used in the control of human and animal parasitic infections by disrupting microtubule function, and via this mechanism, it has demonstrated efficacy against paclitaxel and doxorubicin-resistant cancer cells [12] and in multiple animal models of cancer, including glioma and metastatic TNBC [10, 13, 14]. MBZ and ABZ have been used against CNS pathologies (e.g., neurocysticercosis and cerebral echinococcosis) [15–17], indicating sufficient CNS bioavailability. This has allowed for the fast transition to clinical testing of MBZ in adult high-grade glioma and pediatric glioma [18–20].

The effectiveness of MBZ in animal models of glioma and metastatic TNBC highlights both its potential as an alternative oncologic therapeutic and its potential utility against TNBC CNS metastasis. The identification of promising agents in preclinical CNS models would represent substantial progress in the investigation and treatment of CNS metastases. In the present study, we hypothesized that the tubulin-binding properties of MBZ would counter the migratory capacity of TNBC, reduce active metastatic dissemination (LMD) and, therefore, delay mortality.

## Methods

### Cell culture

Brain-tropic TNBC MDA-MB-231-BR cell line was a kind gift from Dr. J.E. Price (M.D. Anderson Cancer Center, Houston, TX), and was obtained from brain metastases formed after internal carotid (ICA) injection of MDA-MB-231 cells, as described [21]. Brain-tropic luminal A MCF7-BR cell line was established in our laboratory from MCF7 cells through two cycles of ICA injection, selection from brain cell population, and *in vitro* propagation. All cell lines were maintained in Dulbecco's Modified Eagle Medium (DMEM) supplemented with 10% fetal bovine serum and antibiotics, and incubated at 37 °C in humidified air containing 5% CO_2_. The MDA-231-BR and MCF7-BR cell lines were transfected with firefly luciferase to enable later *in vivo* luminescence imaging.

#### High-throughput cytotoxicity assay

The high-throughput screen of the Sum149PT and MCF10a cell lines was conducted as previously described [22]. The viability of MDA-MB-231 and MCF7 cell lines was determined 24 h and 48 h after compound addition, respectively. Cell-Titer Blue assay and Bright-Glow luciferase assay (Promega, WI) were used to assess cell viability. For all assays, the compounds were tested in a 7-point dose response at a final concentration of 20, 10, 5, 2.5, 1.25, 0.625, and 0.3125 μM. We tested the following libraries: LOPAC1280, Microsource Spectrum (2000 compounds), and the Biomol ICCB bioactive (480 compounds) and FDA-approved library (640 compounds).

### Cell viability assay

Cells were seeded at 500–1000 cells/well in 96-well tissue culture plates. MBZ was added to the cells after 16 h incubation at 37 °C. The MTS assay (CellTiter Aqueous One Assay, Promega, WI) was performed on day 7 following drug addition.

### Migration assay

Cell migration was evaluated using transwell migration assay (6.5 mm diameter inserts with 8 μm pore size, polycarbonate membrane (Costar 3422, Corning)). Cells resuspended in serum-free media at a density of 500,000/ml were pretreated with various concentrations of MBZ and 200 μl of cell suspension was added to a top chamber of a 24 well plate. Cells were allowed to settle for 10 min, placed into a lower chamber containing 500 μL of complete media (DMEM-10% FBS) and incubated at 37 °C. At the end of each incubation period, cells were washed twice by gently dipping chamber into a beaker with cold PBS, fixed with 100% methanol for 10 min at -20 °C and stained with crystal violet (0.5% crystal violet in 25% methanol/PBS) at room temperature for 10-15 min. Stain was removed by dipping the chamber in tap water until dye stopped coming off. The membranes were counterstained with 0.3 μM DAPI and rinsed in PBS. Non-migrated cells in the top chamber were rubbed off with a cotton swab stick, making sure that all cells from the edge of the membrane in the top chamber were removed. The membrane was allowed to dry, carefully excised from the well, mounted on a microscope slide, and imaged. Alternatively, the membrane was incubated in 500 μl of 10% acetic acid to dissolve stain with shaking for 10–15 min at room temperature. 150 μL was transferred to a 96 well plate and read OD at 570 nM (Fig. S1).

### Mass spectroscopy detection of MBZ in CSF

Individual analyte and internal standard primary stock solutions (10 mM) were prepared in DMSO. Intermediate stock solutions of MBZ and ABZ were prepared separately in acetonitrile/water (1:1 v/v) buffer. MBZ intermediate stock solution was serially diluted with acetonitrile/water (1:1 v/v) buffer to obtain standard working solutions to generate calibration curves. Calibration curves were prepared by spiking 10 µL of each of the standard working solutions into 50 µL of blank mouse plasma or into 10 µL artificial CSF followed by the addition of 10 µL internal standard solution of ABZ (250 ng/mL for plasma analysis and 25 ng/mL for CSF analysis). Calibration curves were prepared fresh with each set of samples. Calibration curve ranges for MBZ were 4 to 4000 ng/mL for plasma and 0.5 to 500 ng/mL for CSF.

50 µL aliquots of plasma or 10 µL aliquots of CSF were used for analysis. 10 µL internal standard solution was added to 50 µL plasma (or 10 µL CSF) aliquot and mixed by vortexing. 200 µL ice cold solution of methanol/1% acetic acid was added to the sample, samples were vortexed and incubated 1 h at -20 °C to facilitate protein precipitation. After centrifugation, 50 µL (plasma) or 40 µL (CSF) of supernatant was transferred to a new vial, diluted with 25 µL (plasma) or 20 µL (CSF) water, and analyzed by LC–MS/MS.

All analyses were carried out by positive electrospray LC–MS/MS using a Waters Acquity I-class UPLC system with Waters Xevo TQ-XS triple quadrupole mass spectrometer (RRID:SCR_018510). Chromatographic conditions: a Acquity UPLC® BEH C18 2.1 × 50 mm 1.7 µm particle size column (Waters Corp., part number 186002352) was operated at 40 °C at a flow rate 0.25 mL/min. Mobile phases consisted of A: 0.2% formic acid in water and B: 0.2% formic acid in acetonitrile. Elution profile: initial hold at 25% B for 3 min, followed by a linear gradient of 25%-98% in 3 min, hold at 98% for 1 min, equilibrate back to 25% B; total run time was 7 min. Injection volume was 10 µL. Quantitative analysis was done with TargetLynx quantification software (Waters Corp.) using an internal standard approach.

### Infra-red spectra identification of MBZ polymorphs

MBZ was procured from Sigma Aldrich (catalog #M2523, CAS # 31431–39-7). Infrared spectra were measured using a Nicolet iS50 FT/IR spectrometer (Thermo Fisher, MA) using an attenuated total reflectance (ATR) accessory equipped with a diamond ATR crystal.

### Animal model and tumor implantation

All animal studies were approved by the Administrative Panel on Laboratory Animal Care of Stanford University. Unilateral ICA injections were performed in female NuNu mice (Charles River Laboratories) as previously described [23]. Cells were injected in a volume of 20 μl: MDA-MB-231-BR (20,000 cells) and MCF7-BR (50,000 cells). To prevent cell reflux, both the ipsilateral external carotid artery and ipsilateral common carotid artery were ligated. The ICA injection method has also been used previously to model the spread of helminthic cysts [24].

The mice were randomly divided into treatment and control groups once tumor size exceeded 2.5 × 10^5^ photons/sec on bioluminescence imaging (BLI). MBZ was given daily at 50 or 100 mg/kg as oral voluntary ingestion as previously described [25]. These doses have been shown to be effective in murine models of glioma [11, 13, 26]. MBZ emulsion in pure sesame oil was diluted 1:1 with honey and the resulting suspension was diluted 1:1 with 1% hydroxycellulose. Sesame oil was used to augment MBZ enteral absorption [26], and hydroxycellulose was used to prevent MBZ precipitation. Honey increased animal motivation to voluntarily eat the suspension [25]. Control animals received a suspension of hydroxycellulose, pure sesame oil and raw honey. The animals were treated with oral MBZ or control solution daily for the first 21 days and then every 48 h thereafter. Twice-weekly BLI provided quantitative *in vivo* approximates of tumor size, and Kaplan–Meier curves assessed differences in survival. Mice were monitored daily for signs of drug toxicity. For further immunohistochemistry analyses brain tissues were perfused with PBS, dissected and frozen in Optical Cutting Tissue embedding medium.

### Immunohistochemistry

Frozen sections (10 μm) were dried, fixed in 4% paraformaldehyde, quenched in 50 mM NH4Cl and permeabilized by 0.5% Triton X-100. MDA-MB-231-BR3 cancer cells were detected using antibodies against human vimentin (Millipore, CBL202) followed by a secondary Alexa Fluor 488 anti-mouse Fc-gamma subclass 2a specific antibody (Jackson ImmunoResearch Labs, 115–545-206). The following secondary antibodies were used in other applications—Alexa Fluor 488, Alexa Fluor 594, Alexa Fluor 568 (Molecular Probes). MCF7 cells were detected by anti-Pan-cytokeratin antibody (Novus Biologicals, NBP2-33200) or mouse monoclonal anti-human estrogen receptor alpha antibody (Santa Cruz Biotechnology, sc-8002-AF594). Endothelial cells were detected using rat anti-mouse CD31 (BD Pharmingen, 550274) or rat anti-mouse PV1 (BioRad, MCA2539T) antibodies. Cell nuclei were detected with DAPI. Whole skulls were fixed for 72 h using a combined fixation and decalcification protocol (Cal-Ex II, Fisher Scientific, CS511-1D), sectioned and stained with hematoxylin and eosin.

## Results

### Benzimidazoles as potential treatment for migratory cancers, such as TNBC

**Fig. 1:**
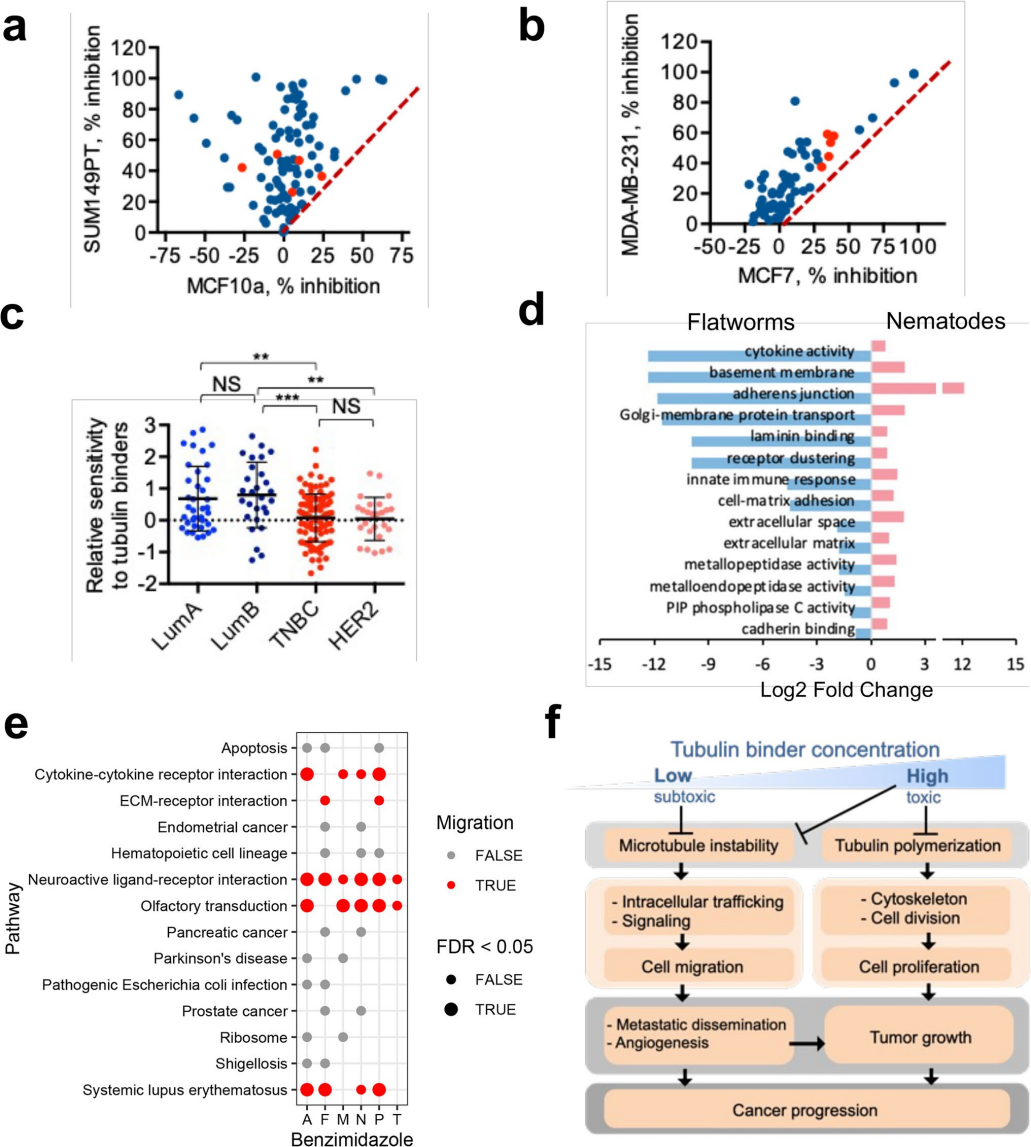
Benzimidazoles as potential treatment for migratory cancers, such as TNBC. **a** Small molecule screen used to identify pharmacologic compounds active against TNBC cell line SUM149PT, but not against a non-tumorigenic breast cell line MCF10a [22]. **b** Benzimidazoles are more effective against metastatic TNBC cell line MDA-MD-231 compared to a metastatic luminal A breast cancer cell line MCF7. (a,b) Diagonal line is placed for agents equally effective against indicated cell lines. Benzimidazoles are labeled in red. **c** Breast cancer cells with high migratory capacity (such as TNBC and HER2 subtype) are more sensitive to tubulin binders. Result of a query of the Genomics of Drug Sensitivity in Cancer (GDSC) database, which included 53 breast cancer cell lines and microtubule inhibitors docetaxel, paclitaxel, vinblastine, and vinorelbine. **d** GO enrichment terms associated with migration are overrepresented in nematodes and underrepresented in flatworms. The data are taken from the study [31] comparing gene expression in nematodes vs flatworms. **e** Disruption of ligand-receptor interactions (important for cell migration and metastasis) represent a common consequence of benzimidazole treatment in mammalian cells (a query of the Drug-Path database [34], The pathways strongly associated with cell migration (red color) are affected by the majority of tested benzimidazoles (albendazole (A), fenbendazole (F), mebendazole (M), nocodazole (N), parmendazole (N), and thiabendazole (T)) and have a low false discovery rate (FDR). **f** Mechanism: Lower concentrations of tubulin binders are needed to inhibit migration. Therefore, migratory cancers should be more responsive to these drugs

Initial small molecule screen performed as part of a high-throughput screen [22] revealed that several benzimidazole drugs selectively inhibited growth of a TNBC cell line, SUM149PT, in comparison to a non-tumorigenic epithelial breast cell line, MCF10a (Fig. 1a). In addition, this pharmacologic class exhibited greater inhibition of growth of another TNBC cell line, MDA-MB-231, compared to a luminal A breast cancer cell line MCF7 (Fig. 1b). To test if selectivity of benzimidazole towards TNBC (Fig. 1a, b) was based on their ability to bind tubulin, we quierried the Genomics of Drug Sensitivity in Cancer (GDSC) database [27] for data on other tubulin binding drugs. We found that sensitivity of TNBC cell lines to clinically relevant tubulin binders from taxane and vinca alkaloid families was significantly higher compared to luminal breast cancer isotypes (Fig. 1c). In addition to TNBC, these tubulin binders significantly reduced the growth of another migratory breast cancer subtype, HER2 (Fig. 1c).

A clue about pathways affected by benzimidazoles in TNBC may come from the helminths’ biology: selective binding of benzimidazoles to helminths’ tubulin has been found to correlate with high efficacy towards helminths and little toxicity towards mammalian cells [28–30]. In addition, benzimidazoles had higher efficacy against parasitic roundworms (Phylum Nematoda) than that of the flatworms (Phylum Platyhelminthes) [28] (Table S1). To find which tubulin-dependent processes might be associated with higher sensitivity to benzimidazoles in nematodes compared to flatworms, we querried a dataset with gene expression data on 56 nematodes and 25 flatworms [31]. Our analysis revealed that Gene Ontology (GO) enrichment terms associated with migration within extracellular matrix (ECM), such as cytokine activity, basement membrane, adhesion, ECM, etc., were overrepresented in nematodes and underrepresented in flatworms (Fig. 1d). These GO terms are known to be associated with invasion and penetration of ECM or other tissue barriers during metastatic dissemination [32]. Of note, the free-living nematode *Caenorhabditis elegans* that has been widely used to model parasitic nematodes, is now being used as an in vivo model for the invadopodia-mediated metastatic cancer invasion through the basement membrane [33].

A subsequent query of the Drug-Path database (http://www.cuilab.cn/drugpath) [34], revealed cell migration as significantly impacted by benzimidazoles in mammalian cells. The biological pathways affected by six benzimidazoles found in the database converged into common pathways of cell receptor-ligand interactions (Fig. 1e, Table S2), and included interactions with cytokines and ECM. Additionally, the gene ontology analysis of genes induced by mebendazole in TNBC cell lines MDA-MB-231 and SUM159 releaved overrepresentation of GO terms (13 out of 38) directly related to migration (Additional File 3 in [14]). Thus, in metastatic TNBC cells, as in the nematodes’ developing larvae, benzimidazoles might target processes necessary for cell migration.

An often overlooked feature of tubulin binders’ action that may provide explanation to their specificity to migratory cancers, such as TNBC, is that the concentrations of tubulin binding drugs that are required to affect cell migration are lower than the concentrations required to trigger cytotoxic effects (Fig. 1f) [35–38]. At low, subtoxic, concentrations tubulin binders inhibit microtubule dynamics and, as a result, the microtubules are not able to remodel in response to demands imposed by changes in cell shape that occur during metastatic cell migration/invasion (Fig. 1f). At high, toxic concentrations, tubulin binders begin to interfere with tubulin polymerization, and as a result inhibit cell division, leading to cell death.

Given that benzimidazoles target migration-associated pathways, have sufficient CNS bioavailability, and are active against TNBC, we hypothesized that these drugs might be particularly effective against TNBC CNS metastasis. We chose to investigate the efficacy of benzimidazoles in our previously described animal model of TNBC brain metastasis with a documented spread to leptomeninges [23].

### Mebendazole (MBZ) as a potential treatment against TNBC LMD

**Fig. 2:**
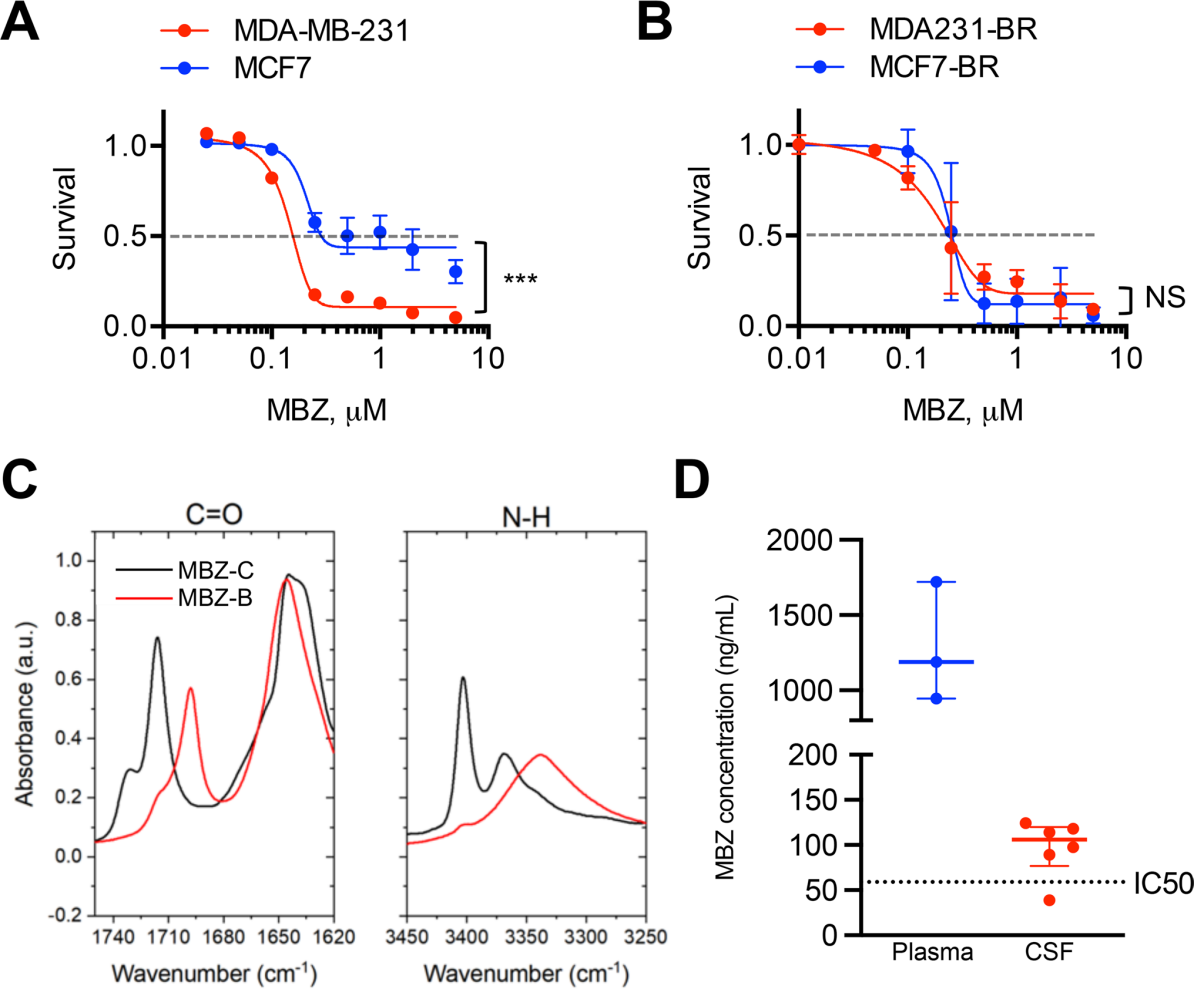
Mebendazole (MBZ) as a potential treatment against leptomeningeal cancer spread. (a, b) Sensitivity to MBZ of TNBC cell line MDA-MB-231 and of hormone receptor positive cell line MCF7 (**a**), and of their brain-tropic derivatives MDA-MB-231-BR and MCF7-BR (**b**). Brain-tropic MDA-MB-231-BR cell line is slightly more resistant to MBZ than the parental cell line MDA-MB-231: MDA-MB-231-BR (IC50 = 0.16 µM), MDA-MB-231 (IC50 = 0.14 µM), MCF7-BR (IC50 = 0.19 µM) and MCF7 (IC50 = 0.19 µM). **c** Infra-red spectra (FT-IR) of MBZ polymorphs revealing the presence of MBZ polymorph C (MBZ-C) and polymorph B (MBZ-B) in the MBZ from Sigma, CAS # 31,431–39-7. **d** MBZ given at an oral dose of 100 mg/kg reaches therapeutic concentrations in the cerebrospinal fluid (CSF) of NuNu mice (median [MBZ] = 105.9 ng/ml ~ 0.36 µM). Plasma MBZ concentrations represent total quantity of MBZ, and CSF concentrations represent free, unbound MBZ. A horizontal line at 59 ng/mL corresponds to the IC50 = 0.20 µM of MBZ. MDA231 = MDA-MB-231. MDA231-BR = MDA-MB-231-BR. Significance: ***, *p* < 0.001, NS = not significant

MBZ is one of the most common benzimidazoles commercially available and has been previously studied in a variety of cancer models. The MDA-MD-231 (IC_50_ = 0.14 µM) cell line exhibited higher sensitivity to MBZ compared to MCF7 (IC_50_ = 0.19 µM), which was consistent with the high-throughput screen results (Fig. 2a). Sensitivity to MBZ was not different between brain-tropic MCF7-BR and MDA-MB-231-BR cells (Fig. 2a,b).

MBZ is commonly manufactured as a mixture of several different polymorphs that are all variably bioavailable and differentially penetrant of the blood–brain-barrier [13]. FT-IR analysis of two different MBZ manufacturers revealed Polymorph C (in black) as the predominate polymorph in one sample (Sigma Aldrich Cat# M2523), as identified by the location of carbonyl and amine functional group absorbance (Fig. 2c). Polymorph B (in red) was the predominate polymorph in the sample from the seco nd manufacturer (Sigma Aldrich Cat# 1,375,502). Since polymorph C was previously described to have superior blood–brain-barrier penetrance and bioavailability in brain, we used MBZ from Sigma Aldrich Cat# M2523 for all subsequent experiments. CSF and plasma samples taken 4 h after oral MBZ administration at a 100 mg/kg dose reached therapeutic concentrations (Fig. 2d), with an average CSF/plasma ratio of 0.09.

### MBZ reduced the migration of TNBC cell lines MDA-MB-231 and MDA-MB-231-BR

**Fig. 3:**
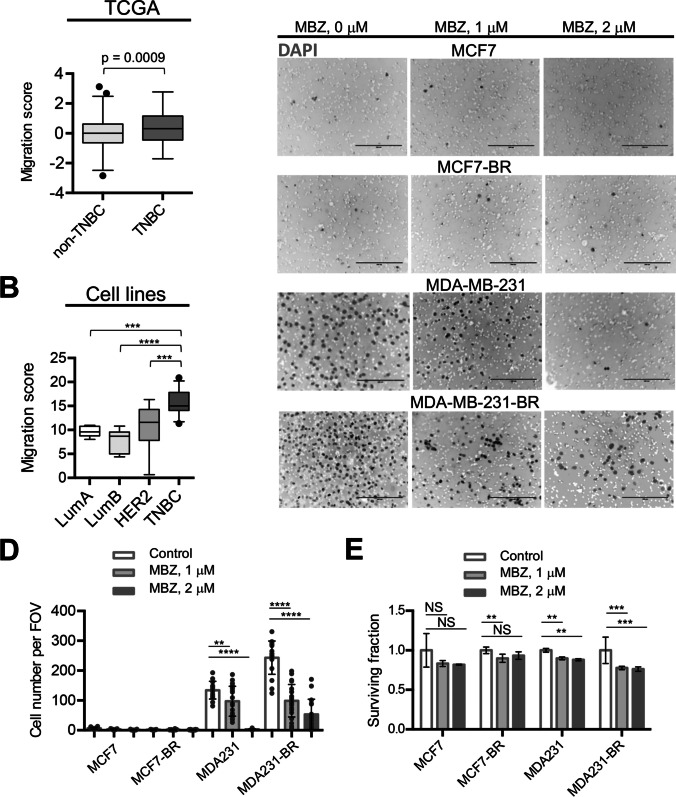
MBZ reduces the migration of TNBC MDA-MB-231 and MDA-MB-231-BR cells. **a**, **b** TNBCs have higher migratory capacity compared to other breast cancer subtypes. Migration scores from Nair et al. [7] were compared among breast cancer subtypes in TCGA patient data (a) and cell lines (b). **c**, **d** MDA-MB-231 and MDA-MB-231-BR have higher migration capabilities than MCF7 and MCF7-BR cells. Migratory capability of MDA-MB-231 cells increases upon acquiring brain-tropic status and is effectively inhibited by MBZ. Neither MCF-7 nor MCF-7-BR migrated significantly during 20 h. (c) Representative inverse fluorescence images of DAPI-stained membranes from Boyden chamber during 20 h. DAPI-stained cell nuclei are shown as dark gray spots in the background of white membrane pores. Scale bar: 200 μm. (d) Quantitation of migration in MCF7, MDA-MB-231, and brain-tropic MCF7-BR and MDA-MB-231-BR cells from (c). The numbers of migrated cells are normalized to survival from (e). **e** Survival during 20 h treatment with MBZ. Plating efficiencies of untreated cell lines were not significantly different. MDA231 = MDA-MB-231, MDA231-BR = MDA-MB-231-BR. FOV = field of view. Significance analysis: ANOVA, **, *p* < 0.01, ***, *p* < 0.001, ****, *p* < 0.0001

Like most benzimidazoles, the mechanism of action of MBZ hinges on selective binding to tubulin of helminths [28] (Table S1). We hypothesized above that benzimidazoles may be effective against migrating cancer cells in the same way they are effective against migrating cells in the developing nematode. *CellToPhenotype* predictor developed by Nair et al. [7] applied to both the TCGA patient tumor samples (Fig. 3a) and to breast cancer cell lines isolated from patient tumors (Fig. 3b) identified TNBCs as the most migratory cancers compared to other breast cancer subtypes. Supporting these results, we showed using in vitro migration assays that the triple-negative MDA-MB-231 cell line (migration score = 14.8 by the CellToPhenotype predictor [7]) exhibited notably greater migration capability than the luminal A MCF7 (migration score = 10.5) cell line (Fig. 3c,d). Importantly, the migratory capability of the MDA-MB-231 cells increased even further upon acquiring brain-tropic status, while the migration of both MCF7 and MCF7-BR remained low (Fig. 3c,d). During the 20-h migration period MBZ effectively inhibited migration of both MDA-MB-231 and MDA-MB-231-BR cell lines (Fig. 3c,d), while cell survival was only modestly affected by MBZ in all cell lines (Fig. 3e). Nair et al. [7] have previously demonstrated that cytoskeletal drugs (such as tubulin binder MBZ) are more effective against cancers with high predicted migration capacity. Consistently, MBZ has been shown to slow down tumor growth and/or prevent metastatic spread of TNBC in animal models using multiple cell lines [14, 39, 40]. To our knowledge, no studies compared MBZ effect on tumor growth and/or survival for distinct breast cancer isotypes. Therefore, we decided to proceed with comparative testing of MBZ and hypothesized that MBZ would be more effective in the MDA-MB-231-BR-based model of CNS metastasis compared to the MCF7-BR-based one.

### MBZ effect in mouse model of TNBC CNS metastasis

**Fig. 4:**
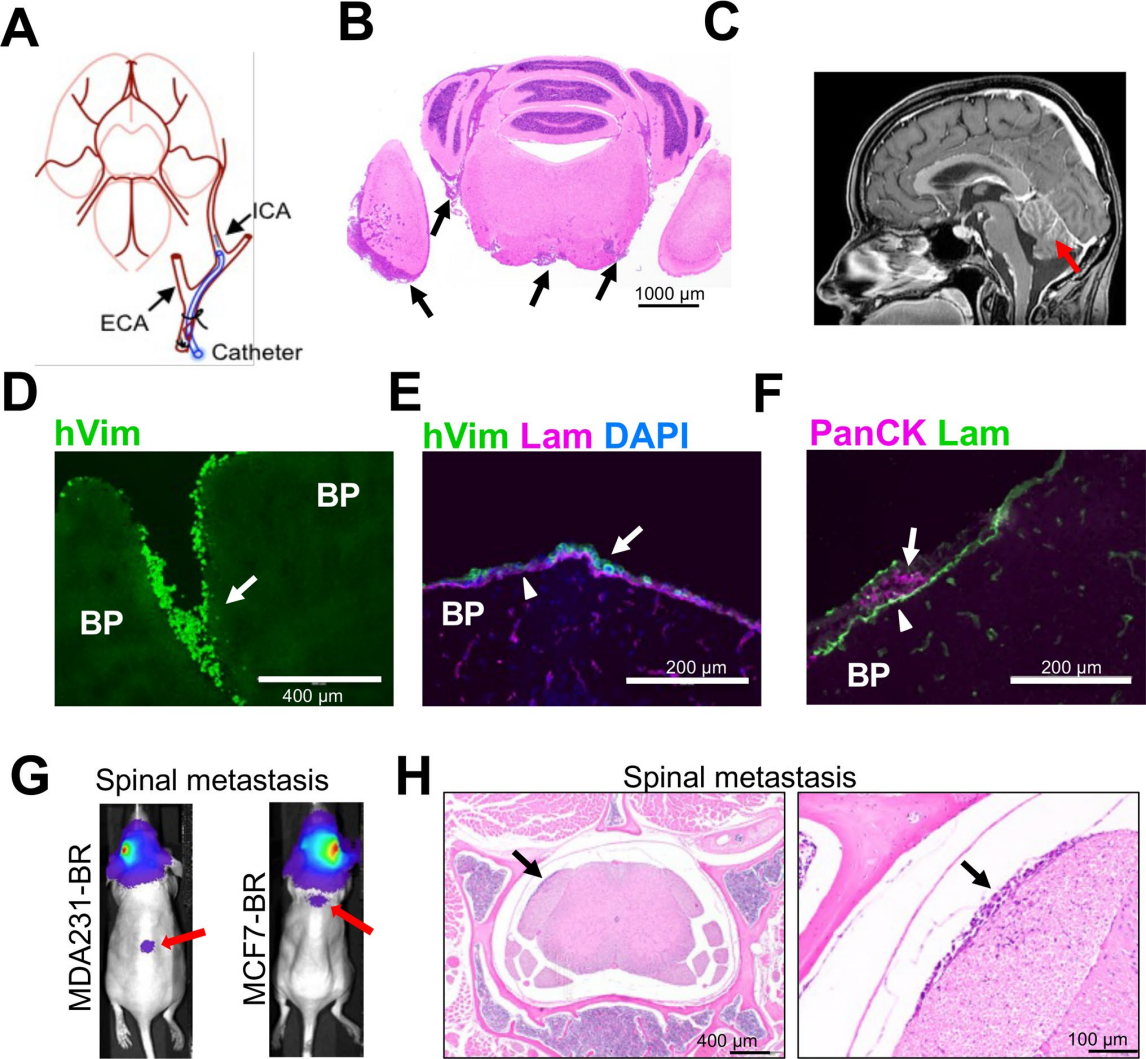
The internal carotid injection (ICA) model of brain metastasis describes leptomeningeal disease (LMD). **a** Schematics of an internal carotid artery injection of tumor cells. **b** H&E-stained section from mouse brain affected by LMD. Arrows point to cancer cells in leptomeningeal space. **c** Patient brain T1W + C MRI sequence shows the anatomical location of LMD (red arrow). (d-h) Immunofluorescence images depict dissemination of neoplastic cells into leptomeningeal space. BP = brain parenchyma. Arrows point to cancer cells in leptomeningeal space. **d**, **e** Vimentin, a marker of epithelial-to-mesenchymal transition, is highly expressed in MDA-MB-231 cells. Antibody against human vimentin (hVim) identifies MDA-MB-231-BR breast cancer cells. (f) Antibody against pan-cytokeratin (PanCK) identifies MCF7-BR breast cancer cells. **e**, **f** Antibody against laminin (Lam) shows the location of pia. **g** Bioluminescence images reveal intracranial disease and spinal dissemination (red arrows). (e) Spinal metastases identified by bioluminescence were verified by subsequent H&E staining. Right panel is a magnified version of a region indicated in the left panel. Black arrows point to the same spinal metastasis in the 4 × image and a magnified (× 20) image

Previous studies demonstrated that migration was a better predictor of breast cancer patient survival than proliferation [7]. The strong inhibitory effect of MBZ on migration of the triple-negative MDA-MB-231-BR (Fig. 3c,d) implied that MBZ might be more effective against active metastatic dissemination. To produce CNS metastasis, cells were injected using the ICA injection method (Fig. 4a), which we have previously shown to result in leptomeningeal spread [23] similar to human LMD (Fig. 4b,c). We chose the ICA method over the more widely used intra-cardiac method to produce CNS-specific metastasis clear of systemic spread which often accompanies the intra-cardiac injection.

To identify the MDA-MB-231-BR cells in mouse brain we used antibody against human vimentin (hVim), a marker of epithelial-to-mesenchymal transition that is highly expressed in the MDA-MB-231 cells [41] (Fig. 4d). MCF7-BR cells were identified by staining with antibodies against pan-cytokeratin (PanCK) or human estrogen receptor (ESR1). Both cell lines produced metastases in leptomeningeal space, as shown by the location of cells relative to the pia basement membrane identified by anti-laminin (Lam) antibody staining (Fig. 4e,f). Parenchymal involvement was different between the cell lines, with MCF7-BR cell line producing more globular and less invasive metastases than the MDA-MB-231-BR cell line (Fig.S2). Presence of parenchymal metastases is consistent with the clinical observations, where in up to 83% of LMD patients, leptomeningeal metastases coexist with parenchymal brain metastases [3, 4, 42]. LMD displayed similar gross heterogeneity in both models, consisting of large bulky metastases, small metastases (< 50 cells/cluster) and a single-cell spread [23] (Fig.S3). Compared to MDA-MB-231-BR, there were very few single-cell/small metastases present in the MCF7-BR population (Fig.S3). In both models, single cell- and small metastasis populations were confined to leptomeningeal space, while large metastases were found both in leptomeningeal and parenchymal compartments. Finally, as in human LMD, in both models some animals developed spinal metastases (Fig. 4g,h).

**Fig. 5:**
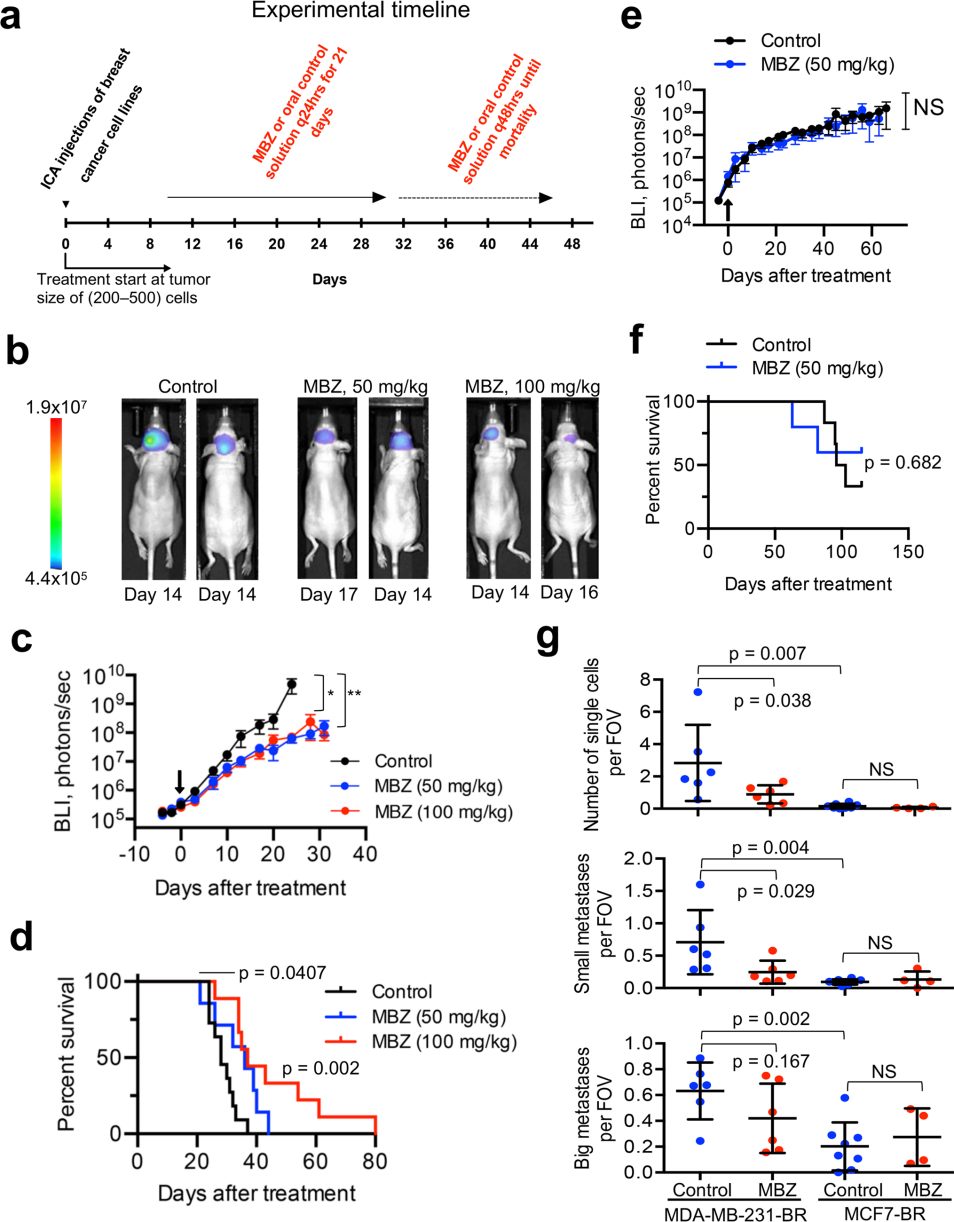
MBZ effectively reduces leptomeningeal dissemination in the TNBC mouse model. **a** Experimental timeline. Tumor size at the start of treatment is between 200–500 cells and corresponds to the BLI signal of (2 – 5) × 10^5^ for MDA-MB-231-BR cell line and (5–10) × 10^5^ for the MCF7-BR cell line. **b** Representative bioluminescence images of MDA-MB-231-BR mice from control and treatment groups (at 50 and 100 mg/kg). See Fig.S5 for original images. **c**, **d** MBZ slows metastatic growth as detected by bioluminescence imaging (c) and improves survival (d) in the MDA-MB-231-BR mouse model. Number of animals per group: control (*n* = 11), 50 mg/kg MBZ (*n* = 7), 100 mg/kg (*n* = 9). **e**, **f** MBZ shows no effect on metastatic growth (e) and survival (f) in the MCF7-BR model. Number of animals per group: control (*n* = 6), 50 mg/kg MBZ (*n* = 5). Experiments in (c, e) were analyzed using repeated measures method. Post hoc pairwise comparisons were performed using a Tukey adjustment for multiple comparisons. Black arrows in (c, e) point to a day of treatment start. **g** MBZ effectively reduces leptomeningeal dissemination (single cell spread and small metastases) in MDA-MB-231-BR model, with no significant effect on big metastases (see Fig. S3 for images). Small metastases are defined as clusters of cells with ≤ 50 cells/cluster without co-option of blood vessels. Data collected from n ≥ 3 mice. Single-cell and small metastatic populations are (20–50)-fold lower in MCF7-BR compared to MDA-MB-231-BR CNS metastasis. Data are mean ± SD. Significance analysis: t-test;*, *p* < 0.05, ***, *p* < 0.001, NS = not significant

The experimental timeline is shown in Fig. 5a. MBZ had a notable effect on the growth of MDA-MB-231-BR tumors at both 50 mg/kg and 100 mg/kg doses (Fig. 5b,c and Fig.S4). No difference in mean BLI signal was observed between the 50 mg/kg and 100 mg/kg groups. Compared to the control, MBZ treatment significantly extended survival in the MDA-MB-231-BR model, with no statistically significant difference in survival between 50 mg/kg and 100 mg/kg groups. In the MCF7-BR model, neither tumor growth (Fig. 5e) nor survival (Fig. 5f) were significantly affected by MBZ treatment (50 mg/kg). Histological examination of brain sections revealed that MBZ effectively reduced metastatic dissemination in the MDA-MB-231-BR model, with a significant effect on single cell- and small metastasis populations (Fig. 5g). MBZ effect on these populations in the MCF7-BR model was not discernible, possibly due to their notably ((20–50)-fold) lower abundance (Fig. 5g and S3b) compared to the MDA-MB-231-BR model (Fig. 5g and S3a). Large metastases were slightly (but not significantly) affected by MBZ in the MDA-MB-231-BR model, with no effect detected in the MCF7-BR model (Fig. 5g). Animal weights were not significantly different between treated and control groups (Fig.S5 and Table S3), suggesting low toxicity.

## Discussion

LMD is a devastating diagnosis for patients with TNBC. Given the universally poor prognosis and limited efficacy of standard-of-care treatments, the investigation of alternative therapies for LMD carries increased importance. Drug repurposing confers obvious advantages in time-to-approval, cost, and safety. We identified MBZ as a drug that through its ability to hamper dissemination of highly migratory TNBC cells in leptomeningeal space, may, therefore, be a suitable candidate for LMD treatment.

MBZ was developed in the 1960s to treat a range of gastrointestinal helminth infections, and it is still one of the most commonly used medications in the world. MBZ safety has been evaluated in 6276 subjects in 39 clinical trials [18]; it can be taken safely in humans at doses as high as 200 mg/kg/day [18, 43, 44] and in rare cases, has been used in humans to treat CNS infections, including neurocysticercosis and echinococcus [17, 45, 46]. Indeed, its relatively small size and lipophilic properties render it an appropriate agent to be repurposed for CNS pathologies [10, 13, 20].

MBZ was successfully tested in multiple preclinical tumor models, including glioma [11, 13, 47] and TNBC [14, 39]. Several active and/or recruiting clinical trials investigating the anticancer effect of MBZ, alone or in combination with other drugs, are currently registered at clinicaltrials.gov [18, 19]. This includes the recent Phase I study conducted by Patil et al. exploring the safety of high-dose MBZ among patients with recurrent glioblastoma [19]. In 11 patients, no dose-limiting toxicity was reached, and the rate of adverse events was low, even when used in combination with temozolomide or lomustine. Other studies, including those involving high-grade glioma, are actively enrolling patients.

Our study is the first effort to test the efficacy of the drug in the treatment of CNS metastasis. We were able to demonstrate that the oral administration of MBZ, at both 50 mg/kg and 100 mg/kg doses, was able to slow tumor growth and increase survival in an aggressive preclinical model of TNBC CNS metastasis. Importantly, our dosing protocol, in which mice voluntarily consumed MBZ in a mix of sesame oil and honey, reached therapeutic concentrations in the CSF, and effectively reduced the leptomeningeal dissemination in MDA-MB-231-BR model. The median CSF concentration was 106 ng/mL for animals treated at the 100 mg/kg dose, which was almost twice the IC_50_ of 56 ng/mL. While CSF concentrations at the 50 mg/kg dose were not measured, animals treated at that concentration still experienced slower tumor growth and increased median survival, both at statistically significant levels, suggesting a robust therapeutic effect.

Bai et al. [13] reported the variability in the efficacy of MBZ across different batches, emphasizing its dependence on the polymorph content. Furthermore, bioavailability and efficacy of MBZ are known to be influenced by intake of fat, which strongly facilitates benzimidazole absorption [48]. These findings suggest that these factors alone could introduce substantial variability in drug efficacy across studies and potentially impact the outcome of clinical trials. Therefore, the dependence of MBZ bioavailability on the drug administration protocol (particularly, fat content) and dosage formulation should be taken into account. For instance, in our study MBZ consisted of highly bioavailable polymorphs B and C, which are optimal for maximum efficacy. Yet, the plasma levels of MBZ in our study, albeit therapeutically significant, were notably lower than those reported by Bai et al. [13]. The observed disparity in plasma MBZ levels compared to the reference study [13] may have resulted from significantly lower fat uptake per MBZ dose in our case.

Our data suggest that MBZ targets cancers with high migratory capacity, and may be particularly effective when these cancers spread into leptomeningeal space, where cancer cell migration could be further enhanced in response to abundant cytokine/chemokine signaling [49]. We’ve shown that among breast cancer subtypes, the TNBCs had the highest migration potential. Consistent with the strong inhibitory effect of MBZ on migration of TNBC MDA-MB-231-BR cells, MBZ extended survival of mice with TNBC LMD. The non-migratory luminal A MCF7-BR cells produced less aggressive CNS metastasis, and were non-responsive to MBZ both in *in vitro* migration assay and in the *in vivo* model.

## Conclusion

In summary, MBZ was demonstrated to be a safe and effective oral agent in an aggressive animal model of TNBC CNS metastasis. MBZ may function by selectively targeting migrating tumor cells. These findings are concordant with previous efforts involving MBZ and CNS pathology and further support the drug’s potential utility to hamper leptomeningeal dissemination.

### Supplementary Information

Below is the link to the electronic supplementary material.

Supplementary file1 (PDF 1529 KB)
