# Supplementary material for: Repurposing mebendazole against triple-negative breast cancer CNS metastasis
Source: J Neurooncol. 2024 Apr 2;168(1):125–38. doi: 10.1007/s11060-024-04654-x (PMC11093727; doi:10.1007/s11060-024-04654-x)
Supplement: Supplementary file 1 — Supplementary file1 (PDF 1529 KB) [file 11060_2024_4654_MOESM1_ESM.pdf]

SUPPLEMENTARY FIGURES

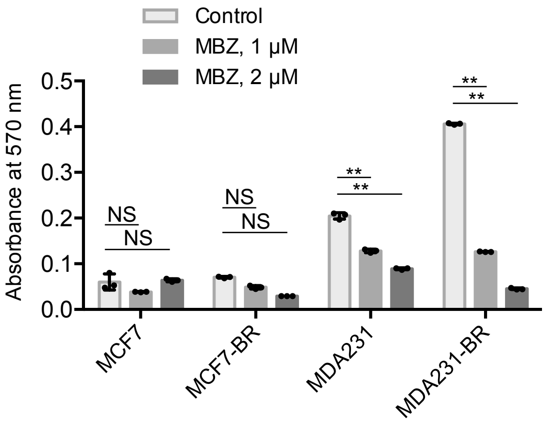

Figure S1. A representative experiment demonstrating anti-migratory effect of mebendazole in breast cancer cell lines. Membranes with migrated MCF7, MDA-MB-231, and brain-tropic MCF7-BR and MDA-MB-231-BR cells were stained with crystal violet (see Materials and Methods and Fig.3). Crystal violet was dissolved in 10% acetic acid, loaded in triplicate wells, and the optical density was measured at 570nm.

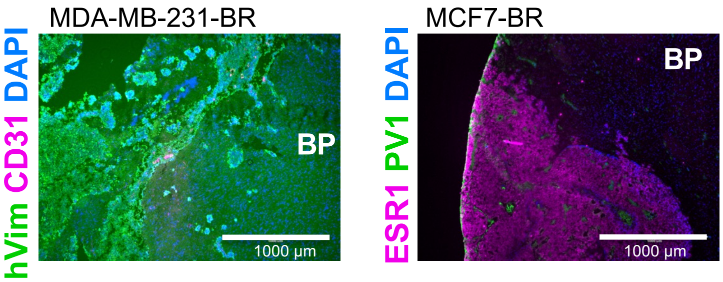

Figure S2. Parenchymal invasion is common in CNS metastasis produced by the ICA model. MDA-MB-231-BR cells have more invasive tumor front than the MCF7-BR cells. Human vimentin (hVim) identifies MDA-MB-231-BR cells, Estrogen receptor (ESR1) identifies estrogen receptor-positive MCF7-BR cells, and CD31 and PV1 are markers of blood vessels.

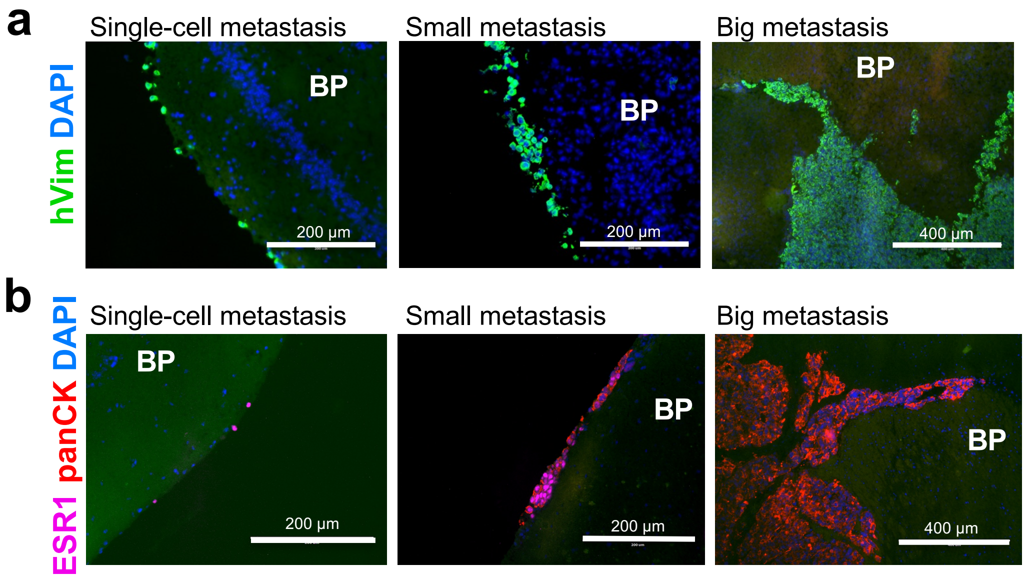

Figure S3. Heterogeneity of leptomeningeal metastasis. (a,b) Leptomeningeal metastasis presents itself as a single-cell spread, small (less than 50 cells per cluster) metastases, and large well vascularized established metastases. (a) Antibody against human vimentin (hVim) identifies MDA-MB-231-BR breast cancer cells. (b) Antibodies against human estrogen receptor (hESR1) and panCK identify MCF7-BR breast cancer cells.

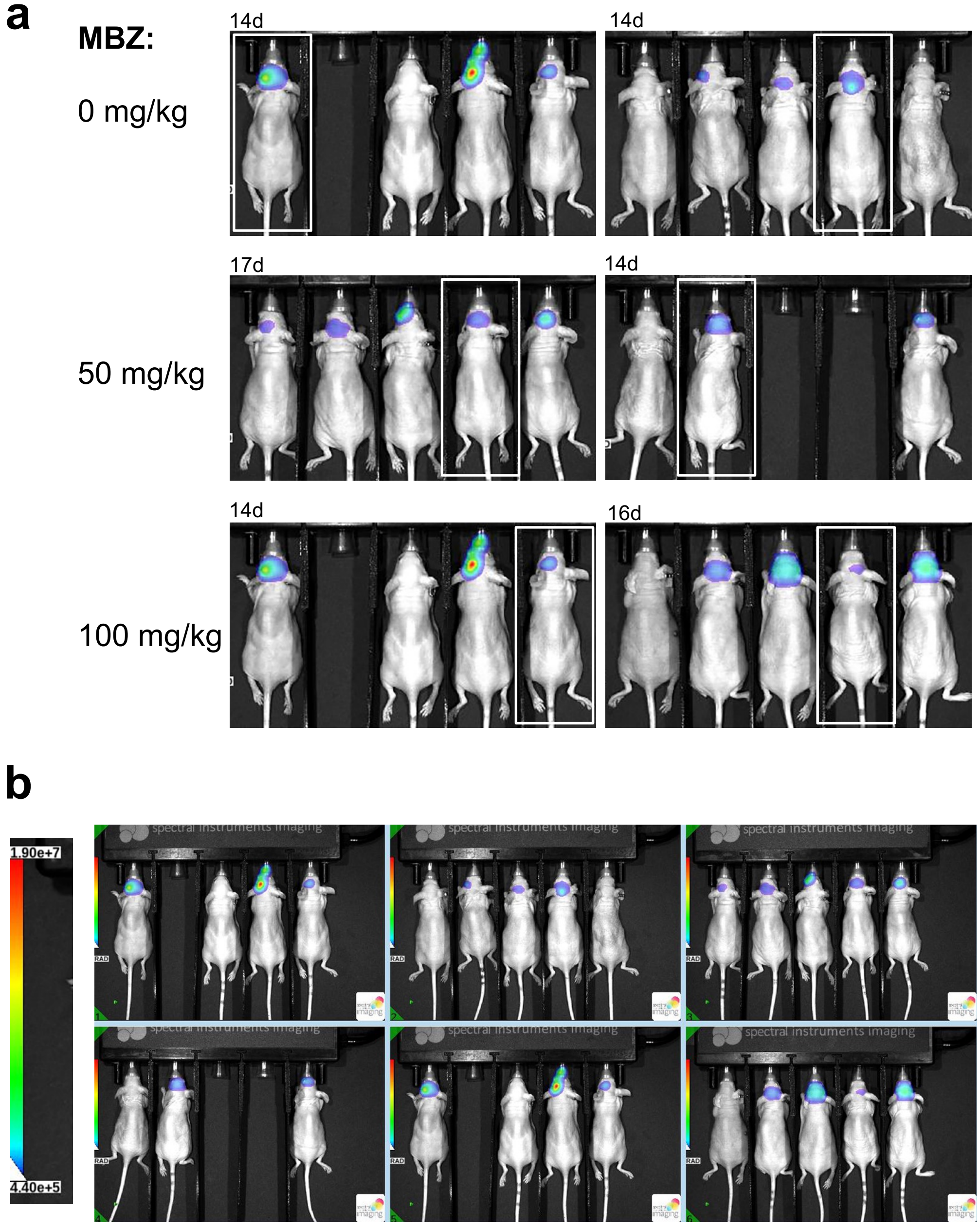

Figure S4. MBZ is effective against TNBC LMD in a mouse model. (a) Original images for Fig. 5B. (b) Screenshot of images from (a) opened in the same window so that the same BLI intensity bar is used for all images.

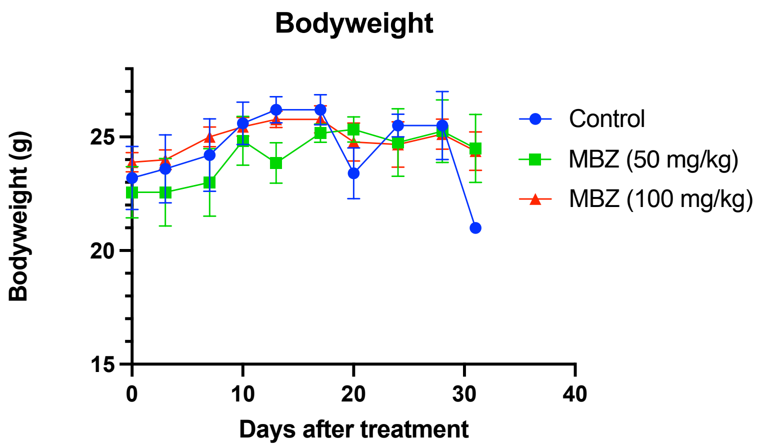

Figure S5. MBZ caused no significant differences in weight trends between control and treated animal cohorts in the MDA-MB-231-BR LMD model.

SUPPLEMENTARY TABLES

Table S1. Efficacy of mebendazole correlates with tubulin binding.

Species	Dose (mg/kg)	[3H]MBZ(pmoles/mg)
**Roundworms**		
Trichostrogylus colubriformis	<12.5	71
Haemonchus contorus	<12.5	63
Ostertagia circumcincta	<12.5	65
Nematodirus spathiger	<15	25
**Flatworms**		
Fasciola hepatica	100	10
Taenia pisiformis	200 (multiple doses)	10
Taeniahydatigena	160-320 (multiple doses)	5
Sheep brain tubulin	>1280	<1

Mebendazole shows higher specificity to tubulin from roundworms compared to flatworms, and very low binding to the mammalian host. Correspondingly, the drug has higher efficacy against roundworms compared to flatworms, and very low toxicity against sheep. Adapted from Lacey, 1988 [28].

Table S2. An output from the query of the Drug-Path database [33] (http://www.cuilab.cn/drugpath).

PathwayID	Pathway	Drug	Pvalue	FDR
hsa04740	Olfactory transduction	albendazole	2.11E-09	1.75E-07
hsa04740	Olfactory transduction	albendazole	3.28E-06	0.000288254
hsa04080	Neuroactive ligand-receptor interaction	albendazole	6.77E-05	0.002808325
hsa04060	Cytokine-cytokine receptor interaction	albendazole	0.00022879	0.00632975
hsa05322	Systemic lupus erythematosus	albendazole	0.0011236	0.023314757
hsa05016	Huntington's disease	albendazole	0.00745275	0.253252212
hsa05012	Parkinson's disease	albendazole	0.0086336	0.253252212
hsa05010	Alzheimer's disease	albendazole	0.01417946	0.256551202
hsa03010	Ribosome	albendazole	0.01457677	0.256551202
hsa04210	Apoptosis	albendazole	0.0178957	0.297068601
hsa05014	Amyotrophic lateral sclerosis (ALS)	albendazole	0.02464665	0.34094536
hsa05131	Shigellosis	albendazole	0.04669957	0.430673857
hsa05130	Pathogenic Escherichia coli infection	albendazole	0.04669957	0.430673857
hsa05012	Parkinson's disease	albendazole	0.0462242	0.430673857
hsa05322	Systemic lupus erythematosus	fenbendazole	4.90E-07	2.99E-05
hsa05322	Systemic lupus erythematosus	fenbendazole	8.98E-07	7.28E-05
hsa04610	Complement and coagulation cascades	fenbendazole	2.98E-06	9.09E-05
hsa04080	Neuroactive ligand-receptor interaction	fenbendazole	0.00120933	0.04897777
hsa05216	Thyroid cancer	fenbendazole	0.00823525	0.133410994
hsa04115	p53 signaling pathway	fenbendazole	0.0080478	0.133410994
hsa05213	Endometrial cancer	fenbendazole	0.00631207	0.133410994
hsa05131	Shigellosis	fenbendazole	0.02823776	0.168415813
hsa05130	Pathogenic Escherichia coli infection	fenbendazole	0.02823776	0.168415813
hsa05212	Pancreatic cancer	fenbendazole	0.02910891	0.168415813
hsa05215	Prostate cancer	fenbendazole	0.02757315	0.168415813
hsa04210	Apoptosis	fenbendazole	0.02910891	0.168415813
hsa03450	Non-homologous end-joining	fenbendazole	0.02412473	0.168415813
hsa05223	Non-small cell lung cancer	fenbendazole	0.02446293	0.168415813
hsa05210	Colorectal cancer	fenbendazole	0.02199168	0.168415813
hsa05222	Small cell lung cancer	fenbendazole	0.02467203	0.168415813
hsa05200	Pathways in cancer	fenbendazole	0.03244986	0.175229264
hsa04640	Hematopoietic cell lineage	fenbendazole	0.03440921	0.408981761
hsa04720	Long-term potentiation	fenbendazole	0.02025426	0.408981761
hsa04512	ECM-receptor interaction	fenbendazole	0.03041982	0.408981761
hsa04630	Jak-STAT signaling pathway	fenbendazole	0.04022771	0.408981761
hsa04740	Olfactory transduction	mebendazole	8.24E-10	7.33E-08
hsa04740	Olfactory transduction	mebendazole	0.00026139	0.01908183
hsa04060	Cytokine-cytokine receptor interaction	mebendazole	0.00304259	0.135395303
hsa05012	Parkinson's disease	mebendazole	0.0060168	0.178498335
hsa03010	Ribosome	mebendazole	0.0098372	0.218877644
hsa04940	Type I diabetes mellitus	mebendazole	0.02335017	0.415633078
hsa05332	Graft-versus-host disease	mebendazole	0.04676474	0.601216667
hsa04080	Neuroactive ligand-receptor interaction	mebendazole	0.04756623	0.601216667
hsa04514	Cell adhesion molecules (CAMs)	mebendazole	0.02256975	0.823795987
hsa04940	Type I diabetes mellitus	mebendazole	0.04895222	1

Table S2, cont. An output from the query of the Drug-Path database [33] (http://www.cuilab.cn/drugpath).

PathwayID	Pathway	Drug	Pvalue	FDR
hsa04740	Olfactory transduction	nocodazole	6.31E-16	5.68E-14
hsa04080	Neuroactive ligand-receptor interaction	nocodazole	1.01E-06	4.54E-05
hsa04060	Cytokine-cytokine receptor interaction	nocodazole	0.00227419	0.068225683
hsa05322	Systemic lupus erythematosus	nocodazole	0.00529059	0.111186688
hsa05212	Pancreatic cancer	nocodazole	0.00741245	0.111186688
hsa05215	Prostate cancer	nocodazole	0.00697352	0.111186688
hsa04010	MAPK signaling pathway	nocodazole	0.00187685	0.153901374
hsa04640	Hematopoietic cell lineage	nocodazole	0.01624723	0.208892935
hsa04710	Circadian rhythm - mammal	nocodazole	0.00669836	0.274632958
hsa04110	Cell cycle	nocodazole	0.04012426	0.451397958
hsa05213	Endometrial cancer	nocodazole	0.04729702	0.472970244
hsa04510	Focal adhesion	nocodazole	0.02271587	0.620900551
hsa04740	Olfactory transduction	parbendazole	5.87E-05	0.005161475
hsa04080	Neuroactive ligand-receptor interaction	parbendazole	0.00027976	0.012309249
hsa05322	Systemic lupus erythematosus	parbendazole	0.00061908	0.013619709
hsa04060	Cytokine-cytokine receptor interaction	parbendazole	0.00060578	0.013619709
hsa04740	Olfactory transduction	parbendazole	0.00514637	0.334514229
hsa04210	Apoptosis	parbendazole	0.03841146	0.636229845
hsa04260	Cardiac muscle contraction	parbendazole	0.04337931	0.636229845
hsa04640	Hematopoietic cell lineage	parbendazole	0.03957903	0.857545614
hsa04512	ECM-receptor interaction	parbendazole	0.03505221	0.857545614
hsa04080	Neuroactive ligand-receptor interaction	tiabendazole	0.02640603	0.476148896
hsa04740	Olfactory transduction	tiabendazole	0.00079978	0.055984687
hsa05221	Acute myeloid leukemia	tiabendazole	0.04738838	0.555013393
hsa04664	Fc epsilon RI signaling pathway	tiabendazole	0.04757258	0.555013393
hsa04370	VEGF signaling pathway	tiabendazole	0.01499683	0.476148896
hsa04912	GnRH signaling pathway	tiabendazole	0.02720851	0.476148896

Table S3. Mebendazole treatment does not significantly affect animal weight. Animal weight loss at late stages of the disease (after day 25) is affected by LMD progression.

mouse #	cage	groups	Day 0	Day 2	Day 3	Day 5	Day 7	Day 9	Day 10	Day 12	Day 14	Day 16	Day 17	Day 19	Day 21	Day 23	Day 24	Day 26	Day 28	Day 30	Day 31	Day 33	Day 35	Day 37	Day 38	Day 40	Day 42
556	5	Control	18		18	19	21		22		24		24		23		23		20								
563	6	Control	21	23		24	26	26		28		29		28		27		27		25		24		18			
564	6	Control	25		25	24	25		27		28		26		24		21										
571	8	Control	19		19	22	23		24		24		25		24		22		18								
574	8	Control	25		25	25	26		26		27		26		25		24		20								
6	10	Control	21		23	25	26		26		28		29		28		26		21								
851	1	MBZ 100mg/kg	25		25	26	26		27		27		27		26		27		26		24		19				
725	2	MBZ 100mg/kg	24	25		25	25	25		27		27		27		26		26		24		19					
722	2	MBZ 100mg/kg	26	25		26		26		27		26		27		25		24		21		16					
551	4	MBZ 100mg/kg	24	25		25	25	25		25		25		24		21		20		21		20		20		21	
573	8	MBZ 100mg/kg	23		24	24	26		26		27		27		27		27		27		27		27		28		28
575	8	MBZ 100mg/kg	23		23	24	24		25		26		27		26		26		26		26		27		28		27
9	10	MBZ 100mg/kg	22		22	23	22		23		24		22		20		19										
724	2	MBZ 100mg/kg	25	25		26		26		26		25		26		26		26		25		19					
567	7	MBZ 100mg/kg	23	22		25		25		25		25		25		26		25		27		28		25			
